# Diagnostic yield of endoscopic markers for celiac disease

**Published:** 2015

**Authors:** DV Balaban, A Popp, F Vasilescu, D Haidautu, RM Purcarea, M Jinga

**Affiliations:** * “Dr. Carol Davila” Central Military University Emergency Hospital, Bucharest, Romania; ** “Carol Davila” University of Medicine and Pharmacy, Bucharest, Romania; *** “Alfred Rusescu” Institute for Mother and Child Care, Bucharest, Romania; **** Tampere Center for Child Health Research, University of Tampere and Tampere University Hospital, Finland

**Keywords:** celiac, endoscopy, markers, diagnosis

## Abstract

**Rationale:** In the setting of open access endoscopy, the recognition of suggestive endoscopic features in the duodenum can select patients with probability of celiac disease (CD). This could add to the current efforts to increase the diagnostic rate of this disease.

**Aim:** The aim of this study was to evaluate the diagnostic accuracy of these markers for CD in an adult population undergoing endoscopy, without a prior serological testing.

**Methods and Results:** Over a period of 3 years, between June 2012 and 2015, all the patients who underwent upper gastrointestinal endoscopy and presented one or more of the endoscopic markers consistent with CD, or those suspected for CD, irrespective of the presence of these markers, were included. Sensitivity, specificity, positive and negative predictive values were calculated for these markers in CD diagnosis. Among the 182 patients, 56.04% were females, with a mean age of 47.6 ± 13.9 years. 20/182 (10.99%) had a final diagnosis of CD. The presence of any endoscopic marker had a high sensitivity (95%) and a negative predictive value (98.41%). Bulb atrophy and reduced folds in the descending duodenum had a low diagnostic accuracy, while scalloping, mosaic pattern and fissures were highly specific for CD (98.77%, 99.38% and 98.77%) and their presence greatly increased the probability of CD, with a positive likelihood ratio of 24.3, 24.3 and 12.15, respectively.

**Discussions:** A wide set of endoscopic markers, including the duodenal bulb, were evaluated in this study. Our results showed that the endoscopy with a careful examination of the duodenum is a sensitive indicator for CD.

**Abbreviations:** CD = celiac disease, GI = gastrointestinal, VA = villous atrophy, NSAID = nonsteroidal anti-inflammatory drug, Sn = sensitivity, Sp = specificity, PPV = positive predictive value, NPV = negative predictive value, AUC = area under the curve, ROC = receiver operating characteristics, WLE = white light endoscopy, NBI = narrow band imaging, tTG = tissue transglutaminase, EMA = anti-endomysial antibodies

## Introduction

Significant efforts have been made to increase the diagnostic rate of celiac disease (CD) in the last decades. Despite the advances in research, the availability of specific serology and point-of-care tests, and the use of case-finding strategies, CD remains much underdiagnosed. Currently, as much as 3 out of 4 CD patients remain undiagnosed [**1**]. This is mainly due to the unrecognition of atypical presentations, lack of widespread screening in high-risk groups and mislabeling as irritable bowel syndrome. An increase in diagnostic rate is needed to prevent CD complications such as anemia, osteoporosis, infertility, or cancer.

In the setting of open access endoscopy and with a great number of procedures undergone for various reasons, the detection of suggestive endoscopic features in the duodenum can select patients with a probability of CD and can aid in increasing the diagnostic rate of the disease. Moreover, while a strategy of routine duodenal biopsies for all symptomatic patients undergoing upper GI endoscopy would certainly be excessive and increase burden on endoscopy and pathology departments, with a low diagnostic yield, one based on high-risk symptoms or endoscopic markers would be more efficient [**2**,**3**]. Recently, a biopsy strategy only for patients with villous atrophy detected while using image enhancement techniques (immersion technique, dye and digital chromoendoscopy, zoom, magnification), has been proposed; however, this would miss patients with Marsh 1 lesions [**4**].

**Fig. 1:**
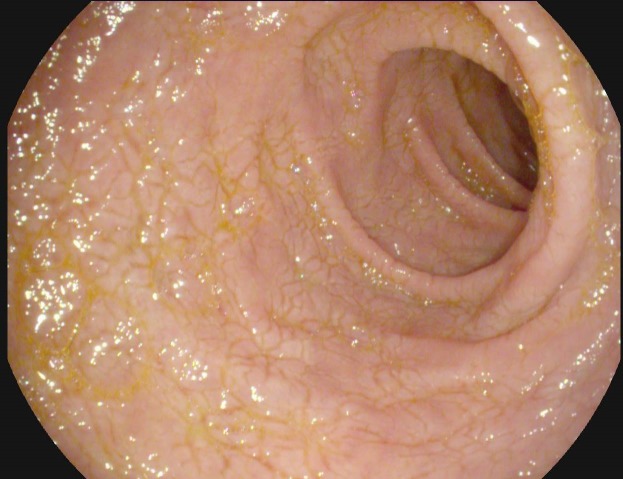
WLE, Fissures (grooves) creating a cracked-mud appearance in the duodenal bulb

**Fig. 2:**
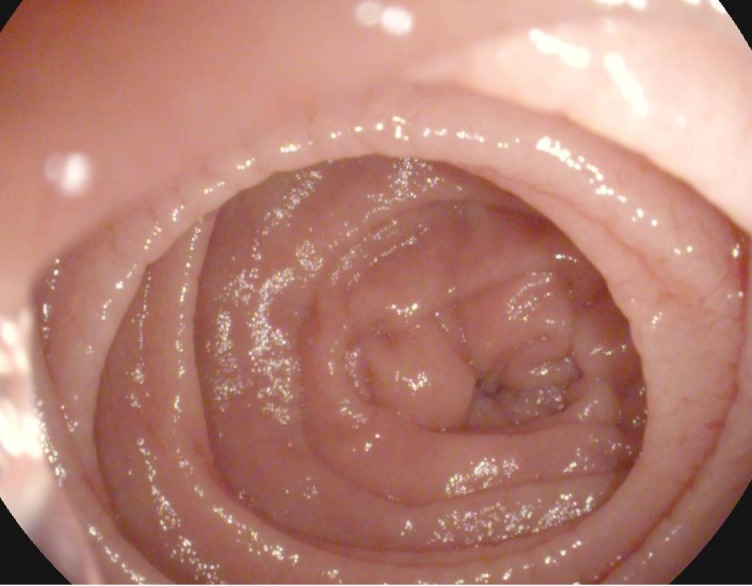
WLE, Scalloping of the Kerckring folds in the descending duodenum

**Fig. 3:**
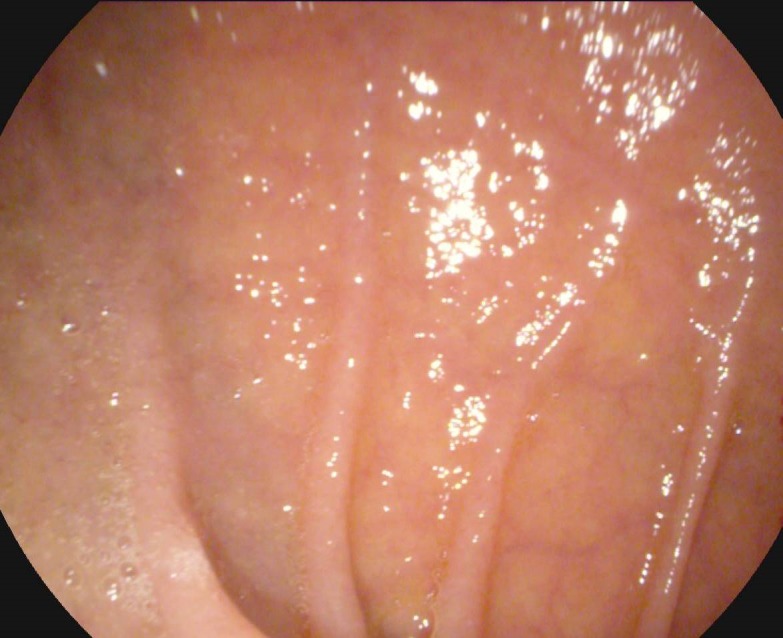
WLE, Atrophy with reduction of duodenal folds

**Fig. 4:**
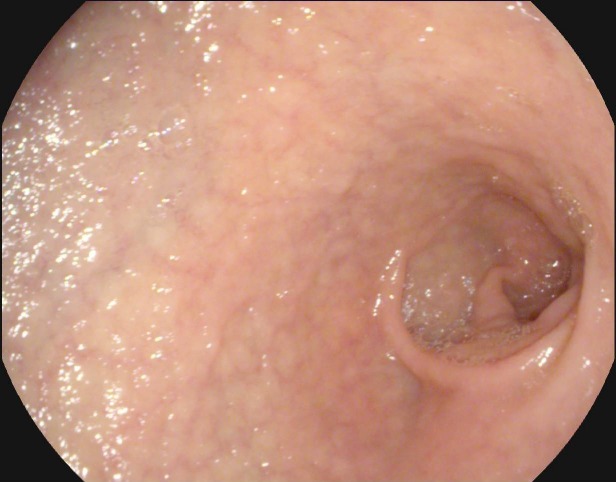
WLE, Atrophy with visible vessel pattern in the duodenal bulb

**Fig. 5:**
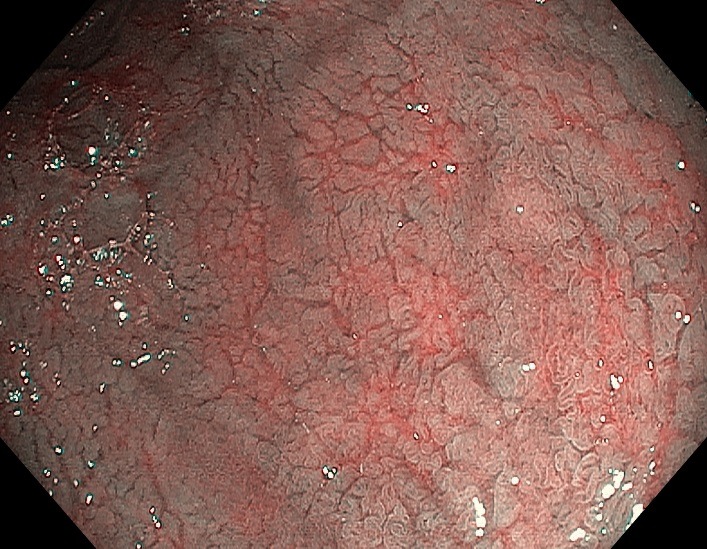
NBI, Proeminent submucosal vessels, and mucosal fissures

Several endoscopic markers have been described in CD: atrophy (with visible submucosal vascular pattern), mosaic or micronodular appearance, presence of fissures (grooves between folds), loss or reduction of folds, flattened or scalloping of Kerckring folds [**5**-**8**] (**Fig. 1**-**5**). 

These markers are usually described in the descending duodenum, with less attention usually being paid to changes in the duodenal bulb [**9**]. Some of these markers can also be associated with other causes (non-celiac) of villous atrophy (VA) such as infectious (Giardiasis, small intestinal bacterial overgrowth, HIV enteropathy), drug-induced (olmesartan, mycophenolate mofetil, methotrexate), autoimmune (autoimmune enteropathy, Crohn’s disease) and others (tropical sprue, collagenous sprue, common variable immunodeficiency, unclassified sprue) [**10**]. 

In the current study, we aimed to evaluate the diagnostic accuracy of these markers for CD in an adult population undergoing upper gastrointestinal endoscopy, with no prior CD serologic workup.

## Materials and Methods

We retrospectively evaluated patients who underwent upper GI endoscopy and presented one or more of the following endoscopic features (atrophy, fissures, mosaic, or nodular pattern in the bulb/ second duodenum, scalloped folds, reduced or absent folds in the second duodenum) or were suspected for celiac disease, irrespective of the presence or absence of these markers. Over a period of 3 years, between June 2012 and 2015, 222 patients altogether met the criteria, but 40 were excluded from analysis because duodenal biopsy or CD serology was either not done or not available. Despite the fact that duodenal erosions have been described in CD [**11**], patients with such changes at endoscopy were excluded because they are more frequently a consequence of peptic or NSAID injury.

Endoscopies were performed by using high definition endoscopes (Pentax, Tokyo, Japan; and Olympus, Tokyo, Japan) by experienced examiners who carefully inspected the duodenum as part of the routine examination. All 182 patients had biopsies sampled from the duodenum and were serologically checked for IgA-tTG antibodies, serum IgA and EMA. CD diagnosis was made according to current available guidelines [**12**,**13**].

Data analysis including sensitivity (Sn), specificity (Sp), positive predictive value (PPV) and negative predictive value (NPV) was carried out by using SPSS Statistics v.17 (SPSS Inc., Chicago, IL) and Epi Info 7.1.5 (CDC, Atlanta, Georgia, USA). Results were expressed as mean ± standard deviation (SD) for continuous variables and proportion for categorical variables. Statistical significance was set at α=0.05. 

## Results

Among the 182 patients included in the final analysis, 56.04% were female, with a mean age of 47.6 ± 13.9 years. 20/ 182 (10.99%) had a final diagnosis of CD. Patient characteristics are presented in **Table 1**.

**Table 1:** Patient characteristics

Demographics	n	%
Gender		
Male	80	43.96
Female	102	56.04
Age		
Mean	47.6 ± 13.9	
Male	46.7 ± 13.4	
Female	48.3 ± 14.4	
Diagnosis		
Celiac	20	10.99
Non-celiac	162	89.01

Endoscopic markers were found in 119 out of the 182 patients (65.38%). The endoscopic findings in the study group are presented in **Table 2**. Among the 119 patients with positive endoscopy, 19 had a final diagnosis of CD. The presence of any endoscopic marker at endoscopy yielded a Sn of 95%, with a modest PPV of 15.97% but very high NPV – 98.41%, which meant that the absence of endoscopic stigmata made a diagnosis of CD very unlikely.

Bulb atrophy and reduction or flattening of Kerckring folds were the most frequent endoscopic markers seen in the study population, but their presence had a low diagnostic yield for CD (Sn 55%, Sp 86.42%, PPV 33.33%, NPV 93.96% for bulb atrophy; Sn 55%, Sp 52.47%, PPV 12.5%, NPV 90.43% for the reduction/ loss of folds in the descending duodenum). 

Scalloping, mosaic pattern and fissures (leading to a cracked-mud appearance) were very characteristic for CD, with 98.77%, 99.38% and 98.77% specificity. The presence of these endoscopic signs greatly increased the probability of CD, with a positive likelihood ratio of 24.3, 24.3 and 12.15, respectively.

**Graph 1:**
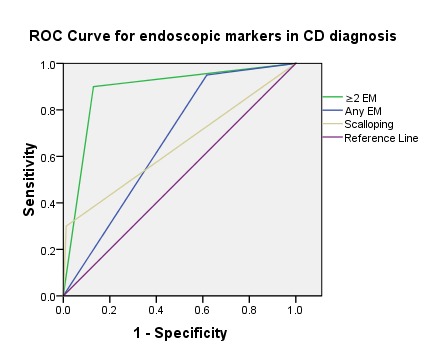
ROC Curve for endoscopic markers (EM) in CD diagnosis

In the CD group, the number of endoscopic stigmata seen at endoscopy was significantly higher than the one in the non-CD group (2.7 vs. 0.77, p<0.01). The presence of more than or equal to 2 endoscopic markers had a good diagnostic performance in predicting CD, with an AUC (area under the curve) of 0.885 (95% CI: 0.803 – 0.967) (**Graph 1**).

**Table 2:** Endoscopic findings

	Endoscopic marker	n	%	CD patients
Duodenal bulb	Atrophy	33	18.13	11
	Mosaic	2	1.09	0
	Fissures	2	1.09	2
	Nodular	13	7.14	6
Descending duodenum	Atrophy	12	6.59	6
	Mosaic	4	2.19	3
	Fissures	5	2.74	3
	Nodular	12	6.59	6
	Scalloping	8	4.39	6
	Reduction, flattening or loss of folds	88	48.35	11
	Any endoscopic marker	119	65.38	19

## Discussion

In the current study, we aimed to evaluate the usefulness of endoscopic markers as predictors for CD in a population with no previous serologic workup.

Several CD endoscopic markers have been described in literature, with variable results regarding their sensitivity and specificity in diagnosing CD, ranging from 6-96.7% and 83-100% respectively [**14**] (**Table 3**). The variability of these results can be explained by the different pre-test probability of having the disease, as seen in comparative studies with low-risk and high-risk populations [**15**,**16**]. With 11% CD diagnosis, our cohort was a high-prevalence one. 

**Table 3:** Endoscopic markers in CD – literature summary

	Markers	Sn (%)	Sp (%)	PPV (%)	NPV (%)	Accuracy
Kasier Y, 2014 [**17**]	Scalloping	48	99	97	-	-
MH Emami, 2008 [**18**]	Fissures	22.2	98.1	14.2	98.8	-
	Scalloping	11.1	98.1	7.6	98.7	-
	Lace pattern	22.2	98.5	18.1	98.8	-
	Reduced folds	11.1	97.3	5.5	98.7	-
	Mild reduction/loss of folds	11.1	98.1	7.6	98.7	-
	Any	77	91	10	99	-
Piazzi L, 2008 [**19**]	Mosaic	61.3	-	65	-	-
	Nodular	65.7	-	75.5	-	-
	Scalloping	64.4	-	64.4	-	-
	Reduction of folds	40.2	-	-	-	-
	Loss of folds	61.5	-	-	-	-
Savas N, 2007 [**20**]	Mosaic	50	50	86.7	13.3	-
	Loss of folds	38.5	25	76.9	5.9	-
	Scalloping	38.5	75	90.9	15.8	-
	Nodular	15.4	50	66.7	8.3	-
Reyes H, 2007 [**16**]	Any (high-risk population)	92.1	93.8	92.1	93.8	93
	Any (low-risk population)	61.1	96.8	40.7	98.6	95.6
Brocchi E, 2002 [**21**]	Any	93.6	99.3	97.3	98.3	98.1
Oxentenko AS, 2002 [**9**]	Loss of folds	47	97	-	-	-
	Mosaic pattern	12	100	-	-	-
	Nodularity	6	95	-	-	-
	Scalloping	6	100	-	-	-
	Any	59	92	-	-	-
Bardella MT, 2000 [**22**]	Any	50	99.6	60	99.4	-
Dickey W, 1999 [**23**]	Any	87.5	100	100	99	-
Niveloni S, 1998 [**24**]	Any	94	100	100	96	-
Magazzu G, 1994 [**25**]	Mosaic pattern or loss of folds	100	99.3	90.9	100	-
Mauriño E, 1993 [**26**]	Any	94	92	84	-	-

In our study population, all the CD patients except for one (19/ 20) had suggestive markers upon endoscopic examination, making upper GI endoscopy a sensitive predictor for CD (Sn 95%). Interestingly, endoscopic markers had a very high NPV (98.41%) for CD, but this should be cautiously interpreted, as exclusion of CD based only on the endoscopic appearance could represent a major pitfall. The problem with endoscopic markers is that they are usually absent in milder forms of disease (Marsh 1, 2 and even 3a) and patchy disease, and for this reason a no-biopsy strategy in normal appearing duodenum cannot be accepted yet [**14**]. Biopsy sampling should always be performed when there is a clinical suspicion, regardless of the presence of endoscopic markers [**14**]. For patchy disease, promising results are coming from the use of advanced endoscopic techniques such as dye staining or digital chromoendoscopy [**14**]. 

Regarding the diagnostic performance of each marker separately, our results showed high specificity for scalloping, mosaic pattern, and fissures, similar to data in literature; their good diagnostic accuracy made some authors conclude that the absence of scallops and grooves exclude VA [**27**]. However, we had a high rate of false negative for the reduction/ loss of folds in the distal duodenum, which led to a low specificity of this marker in the CD diagnosis. This could be explained by the endoscopists’ subjectiveness in the evaluation of the folds, as Niveloni et al. have previously shown that for this marker, the interobserver agreement was the lowest compared to the mosaic pattern or scalloped folds (kappa 0.41, 0.76 and 0.83 respectively) [**24**]. This is also consistent with the results of Reyes H et al. [**16**], who showed that the detection of reduction/ loss of folds only is not a reliable finding unless other markers are also present. 

Considering the previously mentioned limitations of endoscopic markers, there has been some interest to increase their diagnostic accuracy for CD by considering them in addition with other features. Emami MH studied the impact of adding clinical manifestations to endoscopic markers and found that not only did it not improve the Sn, Sp, PPV, and NPV, but also they were all decreased [**18**]. This is probably due to the increasing atypical presentations in adult CD.

There is currently a debate in literature regarding duodenal biopsy sampling for the suspicion of CD: some argue that due to the high NPV of endoscopic markers, biopsy could be avoided in a low-prevalence population if suggestive endoscopic signs are missing on careful examination of the duodenum [**16**], while others state that this would miss infiltrative or hyperplastic enteropathy and advocate for routine duodenal biopsies in all patients, irrespective of the duodenal macroscopic findings [**6**,**28**]. Most published papers agree that the recognition of endoscopic markers by endoscopists is an effective incidental action to improve the CD diagnosis [**29**]. Data from Castro F et al. show that the presence of endoscopic signs is associated with a high probability of diagnosing CD (positive likelihood ratio of 15.6) [**2**]; based on these results, the authors concluded that biopsies should be limited only to high-risk symptomatic patients and those with endoscopic markers. Such an approach is considered wrong by others, which think it is associated with a significant miss rate for CD [**30**].

The best strategy (maximized diagnosis with minimized unneeded biopsies) is probably to combine both pre-endoscopic prediction rules by using clinical-biochemical parameters and endoscopic findings during the procedure [**30**]. The awareness and training of endoscopists in recognizing endoscopic markers is needed to trigger biopsy sampling in cases in which CD diagnosis is not being considered. 

Among our study limitations, we should mention its retrospective nature, but considering the fact that the endoscopists were not set to look for endoscopic markers (as they would have done in a prospective study), this could be thought as a strong point. 

## Conclusions

Although endoscopy is not the first diagnostic tool for the suspicion of CD, the wide access to it creates an opportunity to incidentally diagnose CD. Endoscopy with careful examination of the duodenum is a sensitive indicator for CD, allowing the detection of endoscopic markers which prompt for biopsy sampling. Scalloping, fissures and mosaic pattern are specific endoscopic markers. The high NPV of endoscopic markers should be interpreted with caution, as it can lead to missed CD cases.

**Acknowledgements**

This paper was partly supported by the Sectorial Operational Programme Human Resources Development (SOPHRD), financed by the European Social Fund and the Romanian Government under the contract number POSDRU 141531.

**Sources of funding**

This work was supported by a grant of the Romanian National Authority for Scientific Research, CNDI-UEFISCDI, project number 111/2012.

**Disclosures**

None
